# Interactive Effect of Dietary Gamma-Aminobutyric Acid (GABA) and Water Temperature on Growth Performance, Blood Plasma Indices, Heat Shock Proteins and GABAergic Gene Expression in Juvenile Olive Flounder *Paralichthys olivaceus*

**DOI:** 10.3390/metabo13050619

**Published:** 2023-04-30

**Authors:** Seunghan Lee, Mohammad Moniruzzaman, Nathaniel Farris, Taesun Min, Sungchul C. Bai

**Affiliations:** 1 Aquafeed Research Center, National Institute of Fisheries Science, Pohang 37517, Republic of Korea; shlee5863@naver.com; 2 Department of Animal Biotechnology, Jeju International Animal Research Center, Sustainable Agriculture Research Institute (SARI), Jeju National University, Jeju 63243, Republic of Korea; monir1983@jejunu.ac.kr; 3 Faculty of Biosciences and Aquaculture, Nord University, 8026 Bodø, Norway; nathanielwfarris@gmail.com; 4 Feeds and Foods Nutrition Research Center, Pukyong National University, Busan 48513, Republic of Korea; 5 Department of Animal Biotechnology, Bio-Resources Computing Research Center, Sustainable Agriculture Research Institute (SARI), Jeju National University, Jeju 63243, Republic of Korea

**Keywords:** gamma-aminobutyric acid, water temperature, growth, feed utilization, blood plasma indices, heat shock proteins, GABA-related gene expression, olive flounder

## Abstract

Gamma-aminobutyric acid (GABA) is an important inhibitory neurotransmitter in the central nervous system of living organisms and has the ability to reduce the magnitude of stress in humans and animals. In this study, we evaluated the supplemental effects of GABA on normal and high water temperature based on growth, blood plasma composition as well as heat shock proteins and GABA-related gene expression in juvenile olive flounder. For this, a 2 × 2 factorial design of experiment was employed to investigate the dietary effects of GABA at 0 mg/kg of diet (GABA0 diet) and 200 mg/kg of diet (GABA200 diet) in water temperatures of 20 ± 1 °C (normal temperature) and 27 ± 1 °C (high temperature) for 28 days. A total of 180 fish with an average initial weight of 40.1 ± 0.4 g (mean ± SD) were distributed into 12 tanks, of which, each tank contained 15 fish based on the 4 dietary treatment groups in triplicate. At the end of the feeding trial, the results demonstrated that both temperature and GABA had significant effects on the growth performance of the fish. However, fish fed the GABA200 diet had a significantly higher final body weight, weight gain and specific growth rate as well as a significantly lower feed conversion ratio than the fish fed the GABA0 diet at the high water temperature. A significant interactive effect of water temperature and GABA was observed on the growth performance of olive flounder based on the two-way analysis of variance. The plasma GABA levels in fish were increased in a dose-dependent manner at normal or high water temperatures, whereas cortisol and glucose levels were decreased in fish fed GABA-supplemented diets under temperature stress. The GABA-related mRNA expression in the brains of the fish such as GABA type A receptor-associated protein (*Gabarap*)*,* GABA type B receptor 1 (*Gabbr1*) and glutamate decarboxylase 1 (*Gad1*) were not significantly affected by GABA-supplemented diets under normal or temperature stressed conditions. On the other hand, the mRNA expression of heat shock proteins (*hsp*) in the livers of the fish, such as *hsp70* and *hsp90*, were unchanged in fish fed the GABA diets compared to the control diet at the high water temperature. Collectively, the present study showed that dietary supplementation with GABA could enhance growth performance, and improve the feed utilization, plasma biochemical parameters and heat shock proteins and GABA-related gene expression under the stress of high water temperatures in juvenile olive flounder.

## 1. Introduction

Gamma (γ)-aminobutyric acid (GABA) is a non-proteinogenic amino acid that includes many names such as 4-aminobutanoic acid (preferred by the International Union of Pure and Applied Chemistry, IUPAC) or piperidic acid (though rarely), and is nearly ubiquitous in all forms of life. In a sense, it is easy to take GABA for granted as an important nutritional element, in part, due to the fact that it is abundantly produced de novo within the body, and thus does not have an established dietary requirement. GABA can be found in all kingdoms of life and performs a plethora of functions, and is said to be an important molecule in “inter-kingdom cross-talk”. For years, much of the knowledge about GABA was related to its role in the central nervous system (CNS) and involvement with the tricarboxylic acid (TCA) cycle via the GABA shunt. It was first synthesized in 1883 before which it was known only as a metabolite in plants. It was not until nearly 66 years later that Eugene Roberts and Sam Frankel [1] discovered GABA as an abundant amine in the brain tissues of animals which is synthesized by α-decarboxylation under the action of glutamic acid decarboxylase (GAD) with glutamic acid (Glu) as the substrate [2]. This was over 70 years ago, and since then, our knowledge of GABA’s varied functions and presence has been ceaselessly expanded. The GABA-related receptors such as GABA type A receptor-associated protein (*gabarap*) and GABA type B receptor 1 (*gabbr1*) are two important classes of inhibitory receptors that are activated by GABA in CNS [3,4,5,6,7]. Recently, 23 subunits of the GABA_A_ receptor were identified in zebrafish and suggests that the properties of GABA_A_ receptor subunits are conserved among vertebrates [7]. It has been reported that the GABA_A_ receptor is responsible for balance of excitation and inhibition in neuronal circuits of the olfactory bulb (OB), regulation of total OB output activity and reorganization of odor-encoding activity in fish [3]. On the other hand, the GABA_B_ receptor has three subunits, GABA_B1a_, GABA_B1b_ and GABA_B2_, which are G-protein-coupled receptors for GABA [8]. GABA_B_ has modulatory effect on OB output activity in fish [3]. Thus GABA_A_ and GABA_B_ are differentially involved in multiple functions of neuronal circuits in the OB of fish [3]. In recent years, a great number of studies have been performed to assess the physiological effects that GABA supplementation may have in aquatic species important to the aquaculture industry [9,10,11,12,13]. Although GABA has been utilized for quite some time to induce settling and metamorphosis in abalone [14,15], it has been recently demonstrated to have significant contributions to the growth, feeding behavior, appetite, digestion and immune status of crustaceans and teleost fish [6,16,17,18,19]. In the case of whiteleg shrimp, GABA has been shown to modulate feed intake, growth, antioxidant capacity and disease resistance [10,12]. With regard to teleost fish, it has also been demonstrated that GABA supplementation within an optimal range can generally promote growth, feed intake, antioxidant enzymes, heat shock proteins, disease resistance and modulation of the microbiota [13,20].

Several recent trials have been conducted to investigate GABA supplementation in flat fish, particularly with turbot (*Scophthalmus maximus*) [21] and olive flounder (*Paralichthys olivaceus*) [13]. In a trial by Farris et al. [13], juvenile olive flounder supplemented with GABA (237 mg/kg) demonstrated improved growth, digestive enzyme activities and innate immunity when challenged with the pathogen *Streptococcus iniae.* Similar beneficial results were found by Bae et al. [12] in whiteleg shrimp (*Litopenaeus vannamei*) supplied with a supplementation of GABA at 100 mg/kg including bacterial resistance against *Vibrio alginolyticus*. Thus far, investigations into the effects of GABA in the diet of teleost fish have largely focused on its ability to modulate physiological pathways connected to growth and immune responses under ideal abiotic environmental parameters. To the best of our knowledge, there has not yet been a trial investigating GABA effects on fish exposed to significant variations in temperature. This will be of great relevance since sea temperatures are predicted to continue to warm in the coming years due to climate change [22]. Fish as poikilothermic (i.e., having a body temperature that closely follows the ambient temperature) animals are more vulnerable to rises in water temperature than the other aquatic organisms because most fish have no physiological means of regulating their body temperature, particularly since their gills are highly efficient heat exchangers between the blood and the water [22]. Very few fish species such as tuna, billfishes, and some sharks can use internal heat exchangers to warm particular tissues [23]. Nonetheless, the culture of olive flounder is heavily dependent on flow-through systems utilizing coastal water sources which may make interventions to regulate water temperature in such facilities unsustainable. Thus, the current trial was undertaken to determine the effects that dietary GABA may have on juvenile olive flounder in terms of growth, blood plasma indices and GABA as well as heat stress-related gene expression when exposed to normal and high water temperatures.

## 2. Materials and Methods

### 2.1. Ethical Statement

The experiment was conducted following the guidelines of Institutional Animal Care and Use Committee Regulations, No. 554, issued by the Pukyong National University, Busan, Republic of Korea. Every effort was taken to minimize the suffering of the fish.

### 2.2. Experimental Design

The experiment was a 2 × 2 factorial arrangement of the treatments with two levels of water temperature (normal temperature and high temperature, T0 and T1, respectively) and dietary analytical grade GABA (0 and 200 mg/kg GABA, G0 and G1, respectively). The water temperature of the two groups was maintained at 20 ± 1 °C (normal temperature) and 27 ± 1 °C (high temperature). Prior to the execution of the experiment, the water temperature of the experimental system was increased slowly at a rate of ±1 °C/day using a central heating system to reach the 27 °C for the acclimation of fish to the experimental environment. The basal diet was formulated as shown in Table 1. Two iso-nitrogenous (52% crude protein) and iso-lipidic (11% crude lipid) diets were prepared by supplementing GABA at 0 (GABA0) and 200 mg/kg (GABA200) into the diets (γ-Aminobutyric acid, GABA, ≥99% purity, Sigma-Aldrich, St. Louis, MO, USA). Here, the GABA concentration was chosen based on our recent findings in juvenile olive flounder [13]. For the basal diet formulation, fish meal and soybean meal were used as the major ingredients for protein, fish oil as the major ingredient for lipid, and wheat flour as the major ingredient for carbohydrate. The diets formulation, manufacturing and storage followed the protocols by Bai and Kim [24]. In brief, all the ingredients were mixed together with the addition of oils and water in a feed mixer (HYVM-1214, Hanyoung Food Machinery, Gyeonggi-do, Republic of Korea), and finally produced the feed doughs based on the dietary treatments. The experimental diets were then manufactured by passing the doughs in a pelleting machine (SFD-GT, Shinsung, Gyeonggi-do, Republic of Korea) to form the pellet diets with a diameter of 2 mm which were then dried at room temperature (25 °C) for 48 h. The dried pellets were then made into crumbles, sieved to obtain the desired particle size, sealed in airtight zipper bags and stored at −20 °C until use.

### 2.3. Experimental Fish and Condition

The feeding trial was carried out at the Feeds & Foods Nutrition Research Center (FFNRC), Pukyong National University (PKNU), Busan, Republic of Korea. Olive flounder were brought from a private hatchery (Geoje-si, Republic of Korea). Before starting the feeding trial, the fish were fed with the basal diet for two weeks to become acclimatized to the experimental conditions. At the start of the experiment, 15 olive flounder with an initial weight averaging 40.1 ± 0.4 g (mean ± SD) were distributed into each of the 12 tanks using 2 different temperature control systems (20 and 27 ± 1 °C) with 6 tanks each. The fish were fed at a fixed rate of 1.5~2.0% of wet BW per day twice daily (09.00 and 18.00 h) for four weeks. Filtered seawater was continuously provided in the semi-circulating tanks at a rate of 0.8–1.0 L/min during the study period. Additionally, continuous aeration was ensured in the water tanks in order to maintain saturated dissolved oxygen levels in the fish tanks. The pH of the water in the fish tanks was 7.4 ± 0.2 during the feeding trial. Every day at least 50% of water was exchanged in the tanks to maintain good water quality parameters and a 12 h light:12 h dark photoperiod was used throughout the feeding trial.

### 2.4. Sample Collection

At the end of the 28 days of the feeding trial, the fish were individually weighed from each of the tanks and the average weight gain (WG), specific growth rate (SGR) and percent survival were calculated for the measurement of growth performance of the fish based on the dietary treatments. For the biochemical analyses, three fish from each tank were euthanized with tricaine methanesulfonate (MS-222,100 mg/L, buffered to pH 7.4) for further analyses. Additionally, three fish per tank were sampled for blood plasma analysis as well as liver and brain gene expression. Blood was collected from the caudal vessels of fish with 1 mL heparinized syringes and centrifuged at 1000× *g* for 8 min to collect the supernatant (plasma) for GABA, cortisol and glucose analyses to evaluate the physiological stress response. The samples for liver and brain gene expression were snap-frozen in liquid nitrogen and stored at −80 °C until analysis.

### 2.5. Sample Analyses

The proximate composition of the experimental feeds was determined using the standard methods of AOAC [25]. The moisture contents were determined using a drying oven at 105 °C. Crude proteins were analyzed using the Kjedahl method. Crude lipids were analyzed using soxhlet extraction and the soxhlet system 1046 (Tecator AB, Hoganas, Sweden). Crude ash was determined by combustion at 550 °C. Plasma GABA levels were analyzed using a GABA ELISA assay kit (BioVision, Milpitas, CA, USA) and the cortisol level was analyzed using a cortisol ELISA assay kit (BioVision, Milpitas, CA, USA) according to the manufacturer’s instructions. The glucose levels of the plasma were measured using a chemical analyzer (Fuji DRICHEM 3500i, Fuji Photo Film, Ltd., Tokyo, Japan). Total RNA was extracted from the liver and brain by homogenizing the tissues in TRIzol Reagent (Thermo Fisher Scientific, San Jose, CA, USA). The quantity and quality of the extracted RNA were assessed using a Nanodrop ND-1000 spectrophotometer; the 260/280 ratios were greater than 1.8. The extracted RNA was treated with DNase, then 1 μg of total RNA was reverse-transcribed using the iScript™ cDNA Synthesis kit (BioRad, Hercules, CA, USA). Real-time quantitative PCR was carried out on a CFX96 Real-Time System (BioRad) in a 10 μL total volume reaction using iTaq SYBR Green Supermix (BioRad) and 500 nmol primers according to the protocol provided by the manufacturer. PCR cycling conditions for all genes were as follows: 95 °C for 5 s followed by 55 °C for 30 s over 40 cycles with an initial denaturation step of 95 °C for 3 min. Relative expression levels of the target genes transcripts (*gabarap*, *gabbr1*, *gad1*, *HSP70* and *HSP90*), with *β-actin* as an internal control, were calculated using a CFX manager software version 2.0 (Bio-Rad). The primers used are shown in Table 2. In all cases, each PCR test was performed in triplicate.

### 2.6. Calculation and Statistical Analyses

In this study, using the live weight and feed consumption data, the following indices were calculated:

Weight gain (WG, g/fish) = (g mean final body weight − g mean initial body weight);

Specific growth rate (SGR, %/d) = [(ln mean final body weight − ln mean initial body weight)/number of days] × 100;

Survival (%) = (number of fish at the end of the trial/number of fish at the beginning) × 100;

Feed conversion ratio (FCR) = g total feed consumed/(g final biomass − g initial biomass + g dead fish weight);

Tank mean values (*n* = 3) were used for all statistical analyses. All data were subjected to multi-factorial ANOVA tests using SAS Version 9.4 (SAS Institute, Cary, NC, USA). When a significant main effort or interaction was observed, Tukey’s honestly significant difference (HSD) post hoc test was used to compare the means. Treatment effects were considered significant at *p* < 0.05.

## 3. Results

### 3.1. Effects of GABA and Water Temperature on Growth and Feed Utilization in Olive Flounder

Table 3 shows the effects of temperature manipulation and dietary GABA on growth performance and feed utilization of olive flounder fed the experimental diets for 4 weeks. Mortality was low overall, with no significant differences among the treatment groups (*p* > 0.05). Increasing the temperature from 20 °C to 27 °C significantly reduced final body weight (FBW), growth rate and feed utilization (*p* < 0.05). However, dietary GABA supplementation increased FBW, WG, and feed utilization in terms of reducing FCR (*p* < 0.05) at the high water temperature. There were significant interactions between temperature and GABA on final body weight (FBW, *p* = 0.029), weight gain (WG, *p* = 0.022), specific growth rate (SGR, *p* = 0.034) and feed conversion ratio (FCR, *p* = 0.012) where at the high water temperature the variables were significantly lower than the other treatments. Furthermore, FBW, WG and SGR were significantly lower at the high water temperature compared to the normal temperature treatment groups in fish fed with or without GABA supplemented diets (*p* < 0.05).

### 3.2. Effects of GABA and Water Temperature on Blood Plasma Indices in Olive Flounder

**Figure 1:**
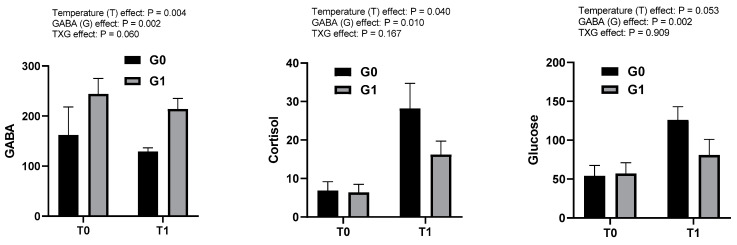
Effects of temperature manipulation and dietary GABA on plasma GABA (pg/mL), cortisol (ng/mL), glucose (mg/dL) contents of juvenile olive flounder. Bars represent mean ± SE (*n* = 6). T0: normal temperature; T1: high temperature; G0: GABA 0 ppm in diet; G1: GABA 200 ppm in diet.

The results of the biochemical assessment of the blood plasma components are presented in Figure 1. Plasma concentrations of GABA, cortisol and glucose were significantly increased with increasing temperature, and decreased with supplementation of GABA in experimental diets (*p* < 0.05). However, the interaction of the main factors (temperature and GABA) failed to have any observable effect on any biochemical assessment in the plasma (*p* > 0.05).

### 3.3. Effects of GABA and Water Temperature on Heat Shock Protein and GABA-Related Gene Expression in Olive Flounder

**Figure 2:**
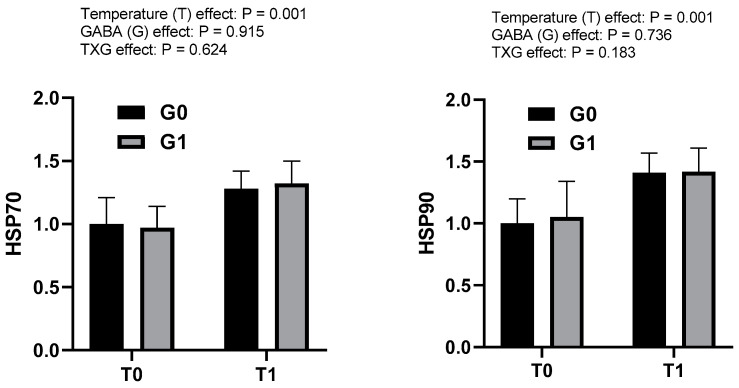
Effects of temperature manipulation and dietary GABA on relative mRNA expression of hsp70 and hsp90 (normalized against β-actin) in the liver of olive flounder juveniles. Bars represent mean ± SE (*n* = 6). T0: normal temperature; T1: high temperature; G0: GABA 0ppm in diet; G1: GABA 200 ppm in diet.

Relative gene expression in the brain and liver tissues of olive flounder by experimental group are presented in Table 4 and Figure 2. Dietary GABA significantly increased *gabbr1* expression, and decreased *gad1* expression (*p* < 0.05) but did not affect that of *gabarap*. However, the expression of *gabbr1* and *gad1* were not significantly different among the experimental groups due to temperature or dietary GABA levels (*p* > 0.05). High temperature resulted in a significant upregulation of *hsp70* and *hsp90* expression in the liver (*p* < 0.05), but dietary GABA had no effect on *hsp70* and *hsp90* expression. The interactions between temperature and dietary GABA were not significant for liver gene expression (*p* > 0.05).

## 4. Discussion

Water temperature is considered as an important parameter in aquaculture production which has direct effects on the performance and economic returns of commercial aquaculture. In recent studies, it has been reported that GABA acts as a neurotransmitter as well as a feed additive that can enhance the performance and alleviate stress conditions in animals [10,26,27]. In this study, we investigated on the effects of GABA on temperature stress conditions in juvenile olive flounder in terms of growth and blood plasma indices as well as heat shock protein and GABA-related gene expression in the liver and brain, respectively. We found that both the GABA and water temperature had significant independent as well as interactive effects on the growth performance of juvenile olive flounder. Fish cultured at a high water temperature without GABA supplementation had significantly reduced FBW, WG and SGR compared to the fish at the normal water temperature. Interestingly, fish supplied with GABA had significantly enhanced FBW, WG and SGR even at the high water temperature in comparison to the fish without supplementation of GABA in the same water conditions. Moreover, there were no significant effects of GABA reflected on the growth at the normal water temperature in fish fed GABA-supplemented or non-supplemented diets. The growth performance data of the present study attributed the water temperature stress attenuation capacity of GABA due to the increased weight of the fish. In addition, dietary GABA and high water temperature showed significant independent and interactive effects on feed utilization in juvenile olive flounder. In this study, fish fed the GABA-supplemented diet had a significantly lower FCR compared to that of fish fed the diet without supplementing GABA at the high water temperature. However, fish with or without GABA supplementation had no significant differences at the normal water temperature. Furthermore, individual feed intake and survival rate in fish irrespective of high or normal water temperature were not affected in the present study. These results indicated that the feed assimilation or conversion of feed to wet weight gain of fish was higher when GABA is supplemented in the diet of juvenile olive flounder. In agreement with our study, El-Nagger et al. [28] reported that dietary GABA at the rate of 100 mg/kg of diet can enhance the growth performance of commercial broilers reared under heat stress conditions. Moreover, Goel et al. [26] recorded higher body weight in chicks supplemented with GABA under thermal stress. Likewise, Xie et al. [10] reported that dietary GABA (150 mg/kg diet) can improve the weight gain in juvenile Pacific white shrimp under ammonia (NH_3_) stress. Furthermore, El-Nagger et al. [28] postulated that GABA-supplemented diets significantly reduced the FCR in Ross broilers under NH_3_ stress which supports the data of the present study on FCR. Food intake in fish is regulated by the central feeding centers of the brain, which receive and process information from endocrine signals from both the brain and periphery [29]. These signals consist of hormones that increase (e.g., orexin; neuropeptide Y, agouti-related peptide (AgRP)) or inhibit (e.g., cocaine and amphetamine regulated transcript (CART), proopiomelanocortin (POMC)) feeding [29,30]. The homeostatic regulation of food intake depends on the release of stimulating (orexigenic neuropeptides) or inhibiting (anorexigenic neuropeptides) hormones that eventually promote or inhibit appetite [31]. Peripheral chemical (e.g., glucose) or endocrine (e.g., gastrointestinal hormones) factors released into the blood cross the blood–brain barrier and have a direct action on the feeding centers thorough peripheral sensory information from the vagus nerve [29]. Under stress conditions, the mechanism of control of food intake in fish are deregulated where appetite-related brain signals do not operate and the expression of appetite-related neuropeptides are changed resulting in a decrease in feed intake in fish. However, the situation can be mediated in part by the corticotropin-releasing factor (CRF), an anorexigenic neuropeptide involved in the activation of the hypothalamic–pituitary–interrenal (HPI) axis during physiological stress responses [31]. In the present study, the increase in fish growth with dietary supplementation of GABA can be verified with the expression of hypothalamus appetite-related factors such as neuropeptide Y, cholecystokinin, orexin, AgRP and ghrelin which can help the high assimilation of feed intake even at high water temperatures [2,29,30]. Dong et al. [32] found that GABA could affect the appetite by regulating the leptin signaling pathway which resulted in alteration of feed intake in Mandarin fish. In an experiment, these researchers confirmed that feed intake in Mandarin fish was significantly increased after GABA intracerebroventricular (ICV) injection (125 μg) within 2 h; however, feed intake at 4 h post-injection showed no significant differences among the tested doses (50, 125, 500 and 2000 μg). Interestingly, Xie et al. [10] postulated that increasing dietary levels of GABA (0, 50, 150, 200 and 250 mg/kg diet) increased the blood insulin and neuropeptide Y levels gradually; however, feed intake in Pacific white shrimp was not significantly increased during an 8-week feeding trial which is in agreement with the results of the present study. The results of the present study indicate that dietary GABA could increase the growth and feed utilization of juvenile olive flounder through the neuronal and hormonal pathways as well as physiological adjustments during high water temperature stress.

Blood plasma indices are an important tool to diagnose the innate immunity or oxidative stress in organisms. As fish are poikilothermic or cold blooded animals, ambient water temperature has a direct effect on the physiology or health status of fish [20]. Cortisol is a steroid hormone that is produced and released from adrenal glands. It is an essential hormone that regulates stress response and blood glucose in animals. Cortisol and glucose are reliable indicators of fish stress which provide a reflection of the severity and duration of the stress response [33]. In teleost fish, the physiological stress response is driven by the activation of two hormonal axes: the brain–sympathetic–chromaffin cells (BSC) axis and the HPI axis [34]. The BSC axis executes the stress response through the rapid rise in plasma catecholamines especially epinephrine and norepinephrine by chromaffin cells which leads to the oxidation of glucose in the plasma and increased energy demand due to stress. On the other hand, the HPI axis is responsible for the increase in levels of plasma glucocorticoids, mainly cortisol, which play an important role in the reallocation and mobilization of energy under stressful conditions [35]. De Abreu et al. [36] reported that fish demonstrate a large response to stress as they possess an HPI which is structurally and functionally similar to the human hypothalamic–pituitary–adrenal axis (HPA). Interestingly, it has been reported that GABA can regulate glucose homeostasis in aquatic animals under fasting stress [28]. Furthermore, GABA can relieve hyperglycemia (high glucose levels) during heat stress and enhance the anti-stress ability of animals [37]. In this study, the results revealed that both the GABA and water temperature had significant independent effects on plasma GABA, cortisol and glucose concentrations; however, no interactive effect between GABA and water temperature was found. It is notable that plasma GABA concentration was increased with GABA supplementation in the diet of fish at normal or high water temperatures. However, the concentration of GABA was lower at the high water temperature compared to that at the normal water temperature. These results clearly demonstrated the strong effect of high water temperature in reducing the plasma GABA concentration in fish. On the other hand, dietary GABA showed its potential effects against high water temperature in terms of depleting plasma cortisol and glucose levels. The results confirmed that dietary GABA reduces the cortisol and glucose in the blood plasma during high temperature stress through regulating the stress response in fish in terms of relieving fish from stress. Consistent with the present study, Jentoft et al. [33] reported elevated levels of glucose and cortisol in the serum in order to handle stress in rainbow trout. However, some studies observed that dietary supplementation of GABA can increase the serum GABA levels as well reduce the serum glucose and corticosterone hormone levels under stress conditions in animals [18,27,38]. These results supported the data of the present study related to GABA, glucose and cortisol levels in juvenile olive flounder under high water temperature stress.

In all vertebrates, the central nervous system (CNS) depends on the balance between stimulatory and inhibitory behaviors of the neurotransmission system (Facciolo et al. 2010). For this, GABA is considered the major inhibitory neurotransmitter in the CNS which activates two classes of receptors, GABA type A receptor-associated protein (*Gabarap*) and GABA type B receptor 1 (*Gabbr1*) [39], in response to stress and feeding behavior in fish [40]. The GABA type A (GABA_A_) and GABA type B (GABA_B_) receptors are commonly known as an ionotropic receptor and metabolic receptor, respectively [2]. On the other hand, glutamate decarboxylase 1 (*Gad1*) enzyme is essential for catalyzing the production of GABA from L-glutamic acid which has an important role in maintaining the stimulatory–inhibitory balance in the CNS [41]. Grone and Maruska [41] opined that *Gad1* is a vertebrate gene which is conserved in teleost fish as a vertebrate animals. It is known that rising water temperatures can reduce the dissolved oxygen (DO) level and increase the oxygen for aquatic animals, as well as elevate the carbon dioxide (CO_2_) levels in water (hypercapnia) which causes serious threat to water breathers like fish [22,42,43]. GABAergic signaling is one of the major pathways that contributes to neuronal survival during anoxia stress by suppressing cellular excitability [44]. Without the protective effects of GABA, brain neurons are incapable of tolerating anoxia and undergo excitotoxicity in terms of excessive glutamate exposure and disruption of the glutamate/GABA ratio that causes cellular swelling, irreversible neuronal injury and eventually cell death [44,45]. It has been reported that without functional GABA_A_ and GABA_B_ receptors, anoxia stress tolerance is lost and neuronal survival is impaired in fish. Therefore, to prevent seizure-like activities in neurons, an intact GABA-mediated inhibitory pathway is required [44]. In the present study, GABA-related gene expression in the brain of juvenile olive flounder such as *Gabarap, Gabbr1* and *Gad1* were analyzed and no significant independent or interactive effect was found in the mRNA expression of *Gabarap* gene in fish fed with or without GABA supplementation at the normal or high water temperature. On the contrary, GABA-supplemented diets showed significant impacts on the *Gabbr1* and *Gad1* gene expression in fish. In this study, the results demonstrated that the mRNA expression of *Gabbr1* was significantly increased with dietary supplementation of GABA in fish. However, the mRNA expression of *Gad1* was significantly reduced in fish supplied with GABA in the diet which might ultimately balance the GABA levels in the CNS of juvenile olive flounder at the high water temperature and relieve the temperature stress. Likewise, Xie et al. [46] reported the positive effects of supplemental GABA in terms of increased mRNA expression of GABAergic receptors such as GABA_A_ and GABA_B_ in the hypothalamic–pituitary–gonadal (HPG) axis of Wenchang chickens. However, these researchers also reported that the GABA_A_ and GABA_B_ receptors showed fluctuations at mRNA levels and variability in the tissues of the HPG over 1–6 weeks of heat stress in chickens. Under normal conditions, fish restore their acid–base balance by increasing hydrogen ion (H^+^) excretion and accumulating bicarbonate ions (HCO_3_^−^) in aquatic environments. A higher level of [HCO_3_^−^] leads to a lower level of chloride ions (Cl^−^) in plasma which occurs due to the action of the GABA_A_ receptor after physiological disruption. When GABA binds to the GABA_A_ receptor, the gate receptor opens and helps to move Cl^−^ from the extracellular medium into neurons which causes an inhibitory function on the neuronal pathway. However, under stress conditions, the concentration of chloride ions is affected by a decrease in Cl^−^ due to an increase in H^+^ excretion so that the binding of GABA, and vice versa, leads to a net Cl^−^ movement out of the neurons into the extracellular medium which causes membrane depolarization and results in an excitatory function in terms of physiological disruption [47]. In the present study, since the high temperature stress did not affect GABA_A_ mRNA expression (*Gabarap* gene), we assumed that the equilibrium potential of Cl^−^ was not significantly changed in the brains of olive flounder. Importantly, extracellular Cl^−^ and HCO_3_^−^ levels are controlled systemically, primarily by exchange at the gills, whereas intracellular Cl^−^ and HCO_3_^−^ levels are controlled by each and every cell, and may vary between nerve cell populations. As a result, the responses of different brain regions and neuronal circuits could be variable including species differences in fish [22]. Goodman and Wong [48] reported that variations in stress responses in organisms are linked to factors ranging from different stress coping styles and sensitivities of neurotransmitter systems. In this study, in contrast to the GABA_A_ receptor, the GABA_B_ receptor (*Gabbr1* gene) was significantly affected by GABA supplementation in the diet of olive flounder which might be attributed to the enhanced feeding efficiency irrespective of the temperature effect on the fish [5]. This result could be due to the increased levels of neuropeptide Y, cholecystokinin, ghrelin and leptin signaling pathway activity as the GABA_B_ receptor is associated with metabolic pathways [29,30,31,40].

Heat stress due to high water temperatures may have adverse effect on the growth, development, and reproduction of animals [46,49]. Heat shock proteins (HSPs) as stress markers are generally heat-inducible gene products such as HSP60, HSP70 and HSP90; they are considered the major stress-related proteins in terms of physical and metabolic as well as oxidative and thermal stress [26,50]. However, HSP70 and HSP90 proteins are highly conserved cellular proteins that are present in fish [51]. The HSP70 protein is responsible for the folding of polypeptide chains, and function as a molecular chaperone to repair denatured proteins. On the other hand, HSP90 is responsible for supporting different components of the cytoskeleton and steroid hormone receptors [51]. As fish are cold blooded animals, their body temperature varies with changes in the surrounding water temperature. Consequently, changes in water temperature lead to the expression of HSPs [20]. Therefore, HSPs are important indices for the adaptability of fish to ambient water temperature. In this study, the mRNA expression of *hsp70* and *hsp90* in the liver of fish fed the GABA-supplemented diets showed no significant independent or interactive effects with water temperature. However, temperature had great effects on the mRNA expression of *hsp70* and *hsp90*, where high water temperature significantly increased the *hsp70* and *hsp90* expression compared to the fish reared at the normal water temperature. These results demonstrated that GABA has no significant effects on liver *hsp70* and *hsp90* expression; however, high water temperatures create a stress on fish through the cellular response of fish. In agreement with the present study, Goel et al. [26] did not find any significant effects of GABA and found that *hsp70* and *hsp90* genes were highly upregulated during embryogenesis in the liver of chicks hatched under circular heat stress. However, Ncho et al. [27] reported that heat stress elevated *hsp70* and *hsp90* gene expression but the supplementation of GABA with thermal manipulation reduced the *hsp90* expression in chicks. Lei et al. [52] reported that higher HSP90 gene expression indicates the enhanced survivability of cells grown in stressed environments. Furthermore, in the present study, the increased expression of *hsp70* and *hsp90* genes in the liver of fish at high water temperatures might be due to their protective effects on cells as well as hormonal manifestations or physiological adjustments [53].

## 5. Conclusions

Taken together, the results of the present study demonstrated that high water temperatures and dietary supplementation of GABA both showed strong and independent as well as interactive effects on body weight, specific growth rate and feed conversion ratio without impacting feed intake and survival in juvenile olive flounder. Moreover, GABA concentrations in blood plasma and GABAergic receptor gene expression in the brain suggested that GABA supplementation can alleviate the temperature stress in fish through neuronal manifestations. In addition, dietary GABA reduced the plasma cortisol and glucose levels which ultimately protect the fish from physiological dysfunction under temperature stress. Furthermore, *hsp70* and *hsp90* gene expression in the liver was highly upregulated under high water temperature conditions but no effect was observed on the growth and survival of fish which might be due to the protective effect of liver cells and the physiological adjustments in juvenile olive flounder.

## Figures and Tables

**Table 1:** Composition of the experimental diets for juvenile olive flounder (% dry matter basis).

Ingredients	Diets	
	CON	GABA
Fish meal (sardine) ^1^	50.0	50.0
Poultry by-product meal ^2^	6.0	6.0
Soybean meal ^2^	8.0	8.0
Soy protein concentrate ^2^	3.0	3.0
Corn protein concentrate ^2^	3.0	3.0
Wheat gluten meal ^2^	1.5	1.5
Wheat flour ^2^	19.2	19.2
Fish oil (menhaden) ^3^	6.0	6.0
Choline chloride (60%) ^2^	0.6	0.6
Vitamin C (Stay C, 35%)	0.2	0.2
Vitamin premix ^4^	1.0	1.0
Mineral premix ^5^	1.0	1.0
Cellulose ^6^	0.5	0.0
GABA ^7^ (40,000 ppm)	0.0	0.5
Proximate analysis (% dry matter basis)		
Moisture	7.3	7.5
Crude ash	10.9	11.1
Crude lipid	10.9	11.1
Crude protein	52.0	51.8

^1^ Suhyup feed Co., Uiryeong, Republic of Korea; ^2^ The feed Co., Goyang, Republic of Korea; ^3^ Jeil feed Co., Hamman, Republic of Korea; ^4^ contains (as mg/kg in diets): ascorbic acid, 300; dl-calcium pantothenate, 150; choline bitate, 3000; inositol, 150; menadion, 6; niacin, 150; pyridoxine · HCl, 15; rivoflavin, 30; thiamine mononitrate, 15; dl-α-tocopherol acetate, 201; retinyl acetate, 6; biotin, 1.5; folic acid, 5.4; cobalamin, 0.06; ^5^ contains (as mg/kg in diets): NaCl, 437.4; MgSO_4_·7H_2_O, 1379.8; ZnSO_4_·7H_2_O, 226.4; Fe–citrate, 299; MnSO_4_, 0.016; FeSO_4_, 0.0378; CuSO_4_, 0.00033; calcium iodate, 0.0006; MgO, 0.00135; NaSeO_3_, 0.00025; ^6^ Sigma-Aldrich Korea, Yongin, Republic of Korea; ^7^ gamma-aminobutyric acid.

**Table 2:** Primers sequence used in real-time qPCR.

Primers	Sense	Sequences (5′-3′)	Product Size (bp)	Accession Number	Annealing Temperature (°C)
*gabarap* ^a^	Forward	AGTGATGAGAGTGTGTATGGG	204	XM_020095803	60
	Reverse	AGAAATGGATGGGAGAAGGG			
*gabbr1* ^b^	Forward	TCCTTTGCCTTTGCCTCTC	161	XM_020104954	60
	Reverse	CCTCGTCGTTGTTGTTGTC			
*gad1* ^c^	Forward	AGCAGGATCGTGGGTTCCCT	105	XM_020089610	60
	Reverse	GAGAAGTCCGTCTCCGTGCG			
*hsp70* ^d^	Forward	CAGTCCAGGCTGCTATCCTCAT	102	AB010871	60
	Reverse	TCATGACTCCACCAGCAGTCTC			
*hsp90* ^e^	Forward	GAGCGAGACAAGGAGGTGAG	101	KY856948	60
	Reverse	CTGGCTTGTCTTCGTCCTTC			
*β-actin*	Forward	CAGCATCATGAAGTGTGACGTG	200	HQ386788.1	60
	Reverse	CTTCTGCATACGGTCAGCAATG			

^a^ *gabarap*: GABA type A receptor-associated protein; ^b^ *gabbr1*: gamma-aminobutyric acid type B receptor 1; ^c^ *gad1*: glutamate decarboxylase 1; ^d^ *hsp70*: heat shock protein 70; ^e^ *hsp90*: heat shock protein 90.

**Table 3:** Effects of temperature manipulation and dietary GABA on growth performance and feed utilization of juvenile olive flounder after 28 days ^a^.

Treatments		IW (g/fish)	FBW (g/fish)	WG (g/fish)	WG (%/fish)	SGR (%/day)	Feed Intake (g/fish)	FCR	Survival (%)
Temperature	Dietary GABA								
Normal Temperature	0	36.8 ± 0.2 ^a^	62.4 ± 0.8 ^a^	25.6 ± 0.7 ^a^	69.6 ± 1.5 ^a^	2.20 ± 0.1 ^a^	28.9 ± 0.2 ^a^	1.13 ± 0.1 ^c^	100 ± 0.0 ^a^
	200	36.8 ± 0.7 ^a^	62.7 ± 0.2 ^a^	25.9 ± 0.5 ^a^	70.6 ± 2.9 ^a^	2.23 ± 0.1 ^a^	29.0 ± 0.3 ^a^	1.12 ±0.1 ^c^	100 ± 0.0 ^a^
High Temperature	0	37.0 ± 0.1 ^a^	58.1 ± 0.5 ^c^	21.1 ± 0.5 ^c^	56.8 ± 1.1 ^c^	1.87 ± 0.1 ^c^	28.6 ± 0.3 ^a^	1.36 ± 0.1 ^a^	95.6 ± 3.1 ^a^
	200	36.9 ± 0.3 ^a^	60.3 ± 0.2 ^b^	23.4 ± 0.2 ^b^	63.5 ± 0.9 ^b^	2.05 ± 0.1 ^b^	28.9 ± 0.5 ^a^	1.23 ± 0.1 ^b^	97.8 ± 3.1 ^a^
**Means of main effect ^a^**									
Temperature	Normal	36.8 ^a^	62.6 ^a^	25.7 ^a^	70.1 ^a^	2.21 ^a^	29.0 ^a^	1.12 ^b^	100 ^a^
	High	36.9 ^a^	59.2 ^b^	22.2 ^b^	60.1 ^b^	1.96 ^b^	28.7 ^a^	1.30 ^a^	96.7 ^a^
GABA	GABA0	36.9 ^a^	60.2 ^b^	23.3 ^b^	63.2 ^b^	2.04 ^b^	28.8 ^a^	1.24 ^a^	97.8 ^a^
	GABA200	36.8 ^a^	61.5 ^a^	24.7 ^a^	67.0 ^a^	2.14 ^a^	28.9 ^a^	1.18 ^b^	98.9 ^a^
**Two-way ANOVA (*p* value)**									
Temperature effect		0.601	<0.001	<0.001	<0.001	<0.001	0.356	<0.001	0.067
GABA effect		0.675	0.010	0.005	0.018	0.018	0.420	0.005	0.500
Temperature × GABA		0.916	0.029	0.022	0.062	0.034	0.752	0.012	0.500

^a,b,c^ Values are means ± SD from triplicate groups of fish (*n* = 3) where the values within a column without a common superscript differ (*p* < 0.05).

**Table 4:** Effects of temperature manipulation and dietary GABA on relative mRNA expression of GABA-related genes (normalized to β-actin) in the brain of juvenile olive flounder ^a^.

Treatments		*Gabarap*	*Gabbr1*	*Gad1*
Temperature	Dietary GABA			
Normal Temperature	0	1.00 ± 0.2 ^a^	1.00 ± 0.3 ^b^	1.00 ± 0.2 ^a^
	200	0.97 ± 0.3 ^a^	1.22 ± 0.3 ^ab^	0.73 ± 0.1 ^a^
High Temperature	0	0.96 ± 0.1 ^a^	1.08 ± 0.1 ^ab^	0.95 ± 0.2 ^a^
	200	0.94 ± 0.1 ^a^	1.49 ± 0.3 ^a^	0.73 ± 0.3 ^a^
**Means of main effect ^a^**				
Temperature	Normal	0.99 ^a^	1.11 ^a^	0.87 ^a^
	High	0.95 ^a^	1.28 ^a^	0.84 ^a^
GABA	GABA0	0.98 ^a^	1.04 ^b^	0.97 ^a^
	GABA200	0.96 ^a^	1.36 ^a^	0.73 ^b^
**Two-way ANOVA (*p* value)**				
Temperature		0.714	0.177	0.783
GABA		0.777	0.017	0.018
Temperature × GABA		0.953	0.446	0.800

^a,b,c^ Values are means ± SD from triplicate groups of fish (*n* = 3) where the values within a column without a common superscript differ (*p* < 0.05).

## Data Availability

The raw data supporting the conclusions of this article will be made available by the corresponding author without undue reservation.
